# The Influence of Long Working Hours, Occupational Stress, and Well-Being on Depression Among Couriers in Zhejiang, China

**DOI:** 10.3389/fpsyg.2022.928928

**Published:** 2022-06-23

**Authors:** Yu Hong, Yixin Zhang, Panqi Xue, Xinglin Fang, Lifang Zhou, Fang Wei, Xiaoming Lou, Hua Zou

**Affiliations:** ^1^Department of Public Health, Hangzhou Normal University, Hangzhou, China; ^2^Occupational Health and Radiation Protection Institute, Zhejiang Provincial Center for Disease Control and Prevention, Hangzhou, China

**Keywords:** couriers, long working hours, depression, occupational stress, well-being, structural equation model, mediating effects

## Abstract

**Objective:**

This study aimed to examine the association between long working hours, occupational stress, depression, and well-being, and to explore the intermediary effect of occupational stress and well-being between working hours and depression among couriers in Zhejiang, China.

**Methods:**

The study used a cluster random sampling method to select 1,200 couriers from mainstream express companies in Zhejiang, China. The data were collected and analyzed using the Core Occupational Stress Scale (COSS) to measure occupational stress, the Patient Health Questionnaire-9 (PHQ-9) scale to evaluate depression, and the World Health Organization five-item Well-Being Index (WHO-5) scale to assess well-being. Structural equation modeling (SEM) was used to test the hypothesized relationship among the variables.

**Results:**

The phenomenon of long working hours (75.1%) was quite common among couriers in Zhejiang, China. Working hours had a direct positive effect on depression (*β* = 0.008, *p* < 0.001) and on occupational stress (*β* = 0.009, *p* < 0.001), and working hours had a negative effect on well-being (*β* = −0.013, *p* < 0.001). Occupational stress had a direct positive effect on depression (*β* = 0.272, *p* < 0.001), but well-being had no significant direct effect on depression. Working hours had an indirect effect on depression through the mediating effect of occupational stress while the mediating effect of well-being was not found.

**Conclusion:**

Long working hours is associated with occupational stress, well-being, and depression. Our results confirmed that working hours, occupational stress, and well-being were strong predicators of depression. Working hours had a significant indirect effect on depression *via* occupational stress. The result of this study showed that decreasing working hours and reducing occupational stress would be effective for couriers to prevent depression. However, more studies are needed to verify the relationship between working hours and depression.

## Introduction

Long working hours is defined as overtime per week beyond regular working hours. There are many jobs that require workers to work overtime, so long working hours is common all over the world. As the International Labor Organization (ILO) said in its report, more than 488 million workers (approximately 7%) had to work over 55 h a week all over the world (Pega et al., 2021). In China, the data from the China Health and Nutrition Survey (CHNS) have shown that the average working time a week for Chinese the population is about 47 h (Nie et al., 2015), which is longer than the national standard of 40 h per week.

Long working hours will not only deprive people of their break time but also cause serious harm to their health. It is known that long working hours have a negative effect on health, such as hypertension (Cheng et al., 2021), cardiovascular diseases (Lin et al., 2018), and diabetes (Baek et al., 2019). In addition, poor mental health such as job burnout (Hu et al., 2016), occupational stress (Lee et al., 2016), and depression (Virtanen et al., 2018) are also associated with long working hours. Therefore, long working hours have become one of the important factors affecting the physical and mental health of work population. However, conclusions of the relevant studies are controversial because the researches on the health effects of long working hours were inconsistent.

In China, with the advent of the digital economy era, e-commerce has developed prosperously, and express delivery industry has shown a rapid development trend. As an economically developed province in the Yangtze River Delta, Zhejiang has developed great express industry, and the number of the courier continues to expand in the recent years. The courier group has become an important part of Chinese labor force. As known to all, due to the features of work, it is common for couriers to work for a long time without a break. They need to complete their work within the stipulated time, so they always have a heavy workload and have no choice but to keep long time working which will lead to poor mental health. Also, other problems, such as poor working environment and monotonous working style all made couriers face extremely high stress, which not only harm their health but also decrease their productivity. It has become an urgent social problem to protect couriers’ physical and mental health. However, research conducted in China for couriers is limited.

Depression is one of the most common mental health disorders. It has shown that long working hours is an important risk factor for depression (Nakata, 2017). Park et al. (2020) showed that the prevalence of stress, depression, and suicide tended to increase with the increase of the working hours, and all three parameters exhibited a linear dose–response relationship with working hours. One study has shown that occupational stress is associated with depression (Park et al., 2014). And the degree of association between depression and occupation stress are not the same according to the occupation (Meltzer et al., 2009). But, few studies explored the relationship between working hours, depression, and occupational stress.

Occupational stress is the process by which workplace psychological experiences and demands in mental and physical health (Zhang et al., 2021). In the current society, inadequate working conditions always lead to occupational stress (Carmona-Barrientos et al., 2020). The mismatch between the individual and workplace such as terrible environment and overtime working are risk factors for occupational stress. Some studies have shown that long working hours will increase the risk of the job stress response (Nash et al., 2010), so occupational stress increases during long working hours.

Well-being is a “subjective experience” of our own quality of life, and it is also an important indicator of individual mental health (Svold et al., 2021). The previous studies found that long working hours have a negative impact on well-being (Song and Lee, 2021), and well-being is associated with depression, by enhancing well-being will alleviate depressive (Sin and Lyubomirsky, 2009). Some studies explored the mediating effect of well-being, it has been proved that occupational stress acts as mechanism in the links between working hours and well-being (Hsu et al., 2019).

In conclusion, former studies have found that long working hours is associated with depression, occupational stress, and well-being, and both of occupational stress and well-being are the influencing factors of depression. So, we tried to link the relationships among working hours, occupational stress, well-being, and depression and hypothesize a double mediator model. Therefore, based on the above analysis, the purposes of this study are: 1. to investigate the current situation of long working hours among couriers in Zhejiang, China; 2. to find whether working hours directly affects depression, occupational stress, and well-being; and 3. to explore whether working hours affects depression through occupational stress or well-being.

## Materials and Methods

### Subjects

The study used a cross-sectional design from September to November in 2021; 1,200 employees from large, medium, and small 30 mainstream express companies were selected in Zhejiang province. The participants were randomly selected from a courier outlet in a random area. The target population comprised of employees aged 18 years or older, and more than 80% of them were in skilled positions (such as solicitation, sorting, transportation, and customer service). The survey was performed through an anonymous self-administered questionnaire survey, all the investigators accepted unified training before. Auditors were assigned to review issues related to quality, such as checking the completeness and accuracy of the completed scale and logical errors. The study was approved by the Medical Ethics Committee of the National Institute of Occupational Health and Poison Control. All of the participants provided informed consent. Finally, 1,161 valid questionnaires were collected with a response rate of 96.8%.

### Variable Measurement

#### Working Hours

Weekly working hours were determined by the answer to the question “How many hours do you usually work on average one week?” Because the working hours of the participants are generally long, in this study long working hours was defined as more than 48 h per week, according to the relevant regulations of the World Labor Organization.

#### Covariate Measures

Information on demographic, economic status, and health behaviors was collected, including age, gender, level of education (≤middle school, high school or college, university, or ≥graduate school), marital status (unmarried, married, separated, widowed, or divorced), monthly income (¥; ≤3,000, 3,000–4,999, 5,000–6,999, 7,000–8,999, 9,000–10,999 or ≥11,000), working age (0–5, 6–10,11–15, >15), shiftwork status (no or yes), drinking (yes or not), smoking status (yes, no or quit smoking), and exercise (moderate exercise at least 30 min/day or not) were also collected using the questionnaire.

### Outcome Variables

#### Depression

We used the Chinese PHQ-9 scale (Patient Health Questionnaire-9) with nine items to evaluate symptoms of depression. It has been proved valid and reliable in Chinese populations (Wang et al., 2014). Items are scored on a 4-point Likert scale from 0 to 3. A total score of ≥10 was defined as having depression, and higher scores indicated higher levels of depression. The reliability Cronbach’s alpha level of the questionnaire was found to be 0.90.

#### Occupational Stress

The occupational stress of the survey was assessed by using the Core Occupational Stress Scale (COSS). It was established as a new occupation stress assessment for Chinese occupational populations by the National Institute of Occupational Health and Poison Control, which has good a reliability and validity (Wang et al., 2020). This questionnaire includes 17 items, which were extracted from the occupational stress measurement items with local Chinese characteristics, and formed through item discrimination test, exploratory factor analysis. It contains four subscales: social support, organization and return, demand and pay, and autonomy, each of which the participants rated using a 5-point Likert scale, and the social support and autonomy total scores are calculated by the reverse scoring items (6-actual score), because the higher score of social support means higher level of social support the participants obtained, higher autonomy scores indicated higher levels of autonomy the participants had at work, both of them are associated with good mental state and negatively correlated with occupational stress. The higher score of organization and return, the lower level of organizational support and return the participants obtained; higher score of requirements and effort, the more demands and efforts the participants required at work, both of them are associated with poor mental health and positively correlated with occupational stress. Total score of 50 ~ 53, 54 ~ 57, and >57 represents mild, moderate, and severe stress levels, respectively, a total score over 50 is defined as occupational stress. The reliability Cronbach’s alpha level of the questionnaire was found to be 0.86.

#### Well-Being

The Well-Being Index (WHO-5) is widely used as a screening instrument to measure subjective well-being all over the world (Topp et al., 2015), which assessed the psychological well-being in the past 2 weeks (Krieger et al., 2014). The items were also rated using a 5-point Likert scale, with a total score below 13 defined as poor well-being. The reliability Cronbach’s alpha level of the questionnaire was found to be 0.87.

### Statistical Analysis

Data were analyzed using SPSS statistics 25.0 and AMOS 24.0. Descriptive statistics were used to examine demographic characteristics of participants. Pearson’s correlation coefficients were calculated for the four variables.

The structural equation model (SEM) can be applied to estimate abstract concepts using measured variables. Cronbach’s alpha was used to test the reliability of the tools: alpha coefficients exceeding 0.7 were interpreted to represent good reliability. The goodness of fit was analyzed using comparative fit index (CFI), normed fit index (NFI), Tucker–Lewis index (TLI), and root mean square error of approximation (RMSEA). A model that meets the following criteria is considered to have a good fit: RMSEA < 0.08, CFI > 0.90, NFI > 0.90, and TLI > 0.90. The SEM used bootstrap maximum likelihood estimation, and bootstrapping was used to test the statistical significance of the indirect and total effects of the model. Two-tailed values of *p* < 0.05 were considered to indicate statistical significance.

## Results

### Participant Characteristics

Table 1 shows the general information for 1,161 couriers in Zhejiang. Our study population was comprised of 1,161 couriers with a mean age of 33.2 years, and the average length of service was 8.13 years. Most of the participants had a high school education. More than half of the participants were married, with a monthly income of 5,000–6,999. Their average working time was 10.25 h, and those who had to work more than 48 h a week accounted for 75.1%.

**Table 1:** Characteristics of participants.

Variables	Category	N	%
Sex	Male	835	71.9
	Female	326	28.1
Age (years)	18–25	206	17.7
	26–30	297	25.6
	31–40	428	36.8
	41–50	191	16.5
	>50	39	3.4
Education level	≤Middle school	265	22.8
	High school	506	43.6
	College	241	20.8
	University	129	11.1
	≥Graduate school	20	1.7
Marital status	Unmarried	301	25.9
	Married	676	58.2
	Separated	104	9
	Widowed	61	5.3
	Divorced and others	19	1.6
Monthly income (¥)	<3,000	56	4.8
	3,000–4,999	363	31.3
	5,000–6,999	398	34.3
	7,000–9,000	214	18.4
	9,000–10,999	86	7.4
	≥11,000	44	3.8
Working age	0–5	564	48.6
	6–10	227	19.6
	11–15	172	14.8
	>15	198	17.0
Shiftwork status	No	758	65.3
	Yes	403	34.7
Drinking	No	414	35.7
	Yes	747	64.3
Smoking	No	488	42
	Yes	551	47.5
	Quit smoking	122	10.5
Regular exercise	No	622	53.6
	Yes	539	46.4
Working hours	≤40	122	10.5
	41–55	258	22.2
	56–70	376	32.4
	71–85	335	28.9
	≥86	70	6

### Correlations Between Study Variables

The correlation for this study is displayed in Table 2. There were significant and positive correlations between working hours, depression (*r* = 0.140，*p* < 0.01), and occupational stress (*r* = 0.326，*p* < 0.01), which showed that more working hours caused higher levels of depression and occupational stress, and the correlation between depression and occupational stress was positive (*r* = 0.121，*p* < 0.01) too. There was negative correlation between working hours and well-being (*r* = −0.294，*p* < 0.01) which showed that increased working hours took poor mental-being. Also, the associations between well-being and occupational stress (*r* = −0.094, *p* < 0.01) and well-being and depression (*r* = −0.142, *p* < 0.01) was significant and negative too.

**Table 2:** Pearson’s correlation coefficients between working hours, depression, occupational stress, and well-being.

	1	2	3	4
1 Working Hours				
2 Occupational Stress	0.326^**^			
3 Depression	0.140^**^	0.121^**^		
4 Well-being	−0.294^**^	−0.094^**^	−0.142^**^	

** *p < 0.001*.

### Reliability and Validity of the Model

Based on the results of EFA, the KMO of this questionnaire was 0.94, which was greater than 0.70 indicating better possibility of factor analysis. The χ^2^ of Bartlett test of sphericity was 17,552.26 (*p* < 0.001). Cumulative explanatory variance explained 64.0%. The Cronbach’s alpha coefficient of factors was 0.78, indicating that the questionnaire has a good reliability.

### Test of the Study Model

**Figure 1:**
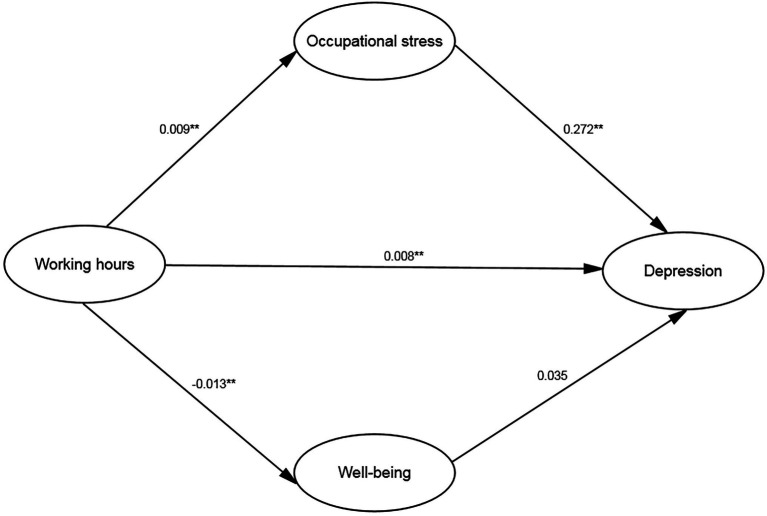
Standardized estimation of the associations of working hours and occupational stress, well-being with depression. **p < 0.01.

The structural model fits the data well: χ^2^ = 340.948; df = 147; χ^2^/df = 2.319; CFI = 0.981, GFI = 0.970, AGFI = 0.962; NFI = 0.967; RMSEA = 0.034; ECVI = 0.366; AIC = 424.976; BIC = 426.440; and CAIC = 679.362. And all of the fit indices suggest an acceptable model fit. Figure 1 illustrates the final output model, showing the correlations and effect paths of research variables.

### Results of Mediation

The path coefficient results of SEM are presented in Table 3. The 95% CI of the estimation of the two-mediation path does not include 0, means that the total effect of working hours on depression was statistically significant, and working hours had significant indirect impacts through occupational stress on depression too. It can be observed that working hours was positively related to depression (*β* = 0.010, *p* < 0.001) and occupational stress (*β* = 0.009, *p* < 0.001), but negatively correlated with well-being (*β* = −0.013, *p* < 0.001). Occupational stress had a direct positive effect on depression (*β* = 0.272, *p* < 0.001). The path coefficient between well-being and depression is not significant (*β* = 0.035, *p* = 0.128), indicating that well-being had no direct impact on depression.

**Table 3:** Significance test of the mediating test.

Model Pathways	Estimated	95% CI
**Total effects**		
Depression←Working hours	0.010	0.009–0.012
Well-being←Working hours	−0.013	(−0.016)–(−0.011)
Occupational stress←Working hours	0.009	0.007–0.011
Depression←Well-being	0.035	(−0.015)–0.079
Depression←Occupational stress	0.272	0.188–0.354
**Direct effects**		
Depression←Working hours	0.008	0.009–0.012
Well-being←Working hours	−0.013	(−0.016)–(−0.011)
Occupational stress←Working hours	0.009	0.007–0.011
Depression←Well-being	0.035	(−0.015)–0.079
Depression←Occupational stress	0.272	0.188–0.354
**Indirect effects**		
Depression←Working hours	0.002	0.001–0.003

As shown in Table 4, in the mediation analysis, analyses of total indirect effects indicated that occupational stress and well-being mediated the relationship between working hours and depression (*β* = 0.002, 95% CI: 0.001–0.003, *p* = 0.001). Working hours was found to have a significantly positive indirect effect through occupational stress on depression. However, well-being had no mediate effect [*β* = 0.000, 95% CI: −(0.01)–0.01, *p* = 0.153], which showed that working hours had no indirect effect on depression through the mediating effect of well-being.

**Table 4:** Significance test of every mediating pathway.

Model Pathways	95% CI
Indirect effect1: Depression←Well-being←Working hours	−0.001-0.001
Indirect effect2: Depression←Occupational stress←Working hours	0.002–0.003

## Discussion

In this study, the prevalence of long working hours among participants was 75.1%. In China, because of the developed express delivery industry, couriers are more prone to working overtime. At the same time, this study also found that the detection rate of occupational stress among express delivery workers in Zhejiang Province was 32.4%, and the detection rate of depression was 32.5%, which was higher than the results of the epidemiological survey of the general population in China (Lee et al., 2009). Therefore, more attention should be paid to the long working hours among couriers.

Occupational stress and well-being were found to be significant influencing factors of depression. So, the study integrated four variables in one model, including working hours, depression, occupational stress, and well-being. Based on the results from SEM, working hours had direct and indirect effects on the depression of couriers through the mediating roles of occupational stress. Long working hours will directly increase the work requirements of workers and increase the probability that being exposed to sources of occupational stress, which will lead to occupational stress. Lee et al. (2016) have found that working more than 60 h a week is more likely to cause occupational stress. One study in Japan has found that, compared to those who worked without long working hours (less than 9 h a day), in the association between working hours and stress response, the odds ratio would increase among those who had long working hours (Matsushita and Yamamura, 2022).

The model also showed long working hours negatively influenced well-being, which was the same as previous studies (Chang et al., 2020). In China, a study found that long working hours were significantly associated with depression and poor well-being among employees (Li et al., 2019), which proposed that long working hours will decrease the free time for workers so that they have no time to relax which leads to poor well-being. Also, long working hours which could lead to a shortage of sleep, activation of the hypothalamic–pituitary–adrenal axis (HPAA) due to sleep deprivation, may also be detrimental to well-being. It is necessary to keep couriers’ working hours within a reasonable range to protect their mental health and reduce the incidence of various mental illnesses.

In this study, we found that the severity of occupational stress increased as the depression severed, which was the same as the previous study (Oenning et al., 2018; Romswinkel et al., 2018; Yörük and Güler, 2021). Previous studies have indicated that someone who had occupational stress may have a higher risk of mental diseases like depression (Yoshizawa et al., 2016), especially during the time during the outbreak of COVID-19 in China, which showed that depression among workers is directly related to stressors in the work environment (Gao et al., 2012). In conclusion, the relationship between depression and occupational stress has been proved and the mechanism between them is needed to be explored in the future, but what we can be sure of is that depression can be effectively prevented by reducing the occurrence of occupational stress.

The working environment often has an impact on the mental health of workers (Gao et al., 2014). According to the results, well-being researches had no direct effect on depression, nor did it mediate between working hours and occupational stress which is inconsistent with the former (Fava et al., 2017; White et al., 2018). But, the correlation between well-being and depression was found. As a meta-analysis shown, depression status was related to well-being (Sin and Lyubomirsky, 2009) and well-being scores were lower in depressed participants (Edmondson and MacLeod, 2015). Other studies have also reported significant correction between depression and well-being (Spielmans and Gerwig, 2014; Muñoz, 2019). So, improving mental well-being will bring more benefit to depressed patients (Steinert et al., 2016). Also, in Korea, Choi et al. (2021) have found significant partial mediation pathways between working hours and depression through mental well-being. In the future, we may try to increase the sample size to reconfirm this result.

However, the effect of working hours and occupational stress on depression was affirmed, and we found that the relationship between working hours and depression was mediated *via* occupational stress among couriers in Zhejiang, China. A cross-sectional study among employees in Korea has proved the mediation effect of occupational stress between working hours and depression because social support was a key factor affecting occupational stress related to working hours and depression (Yoon et al., 2018). In America, social support at work was proved to related to be depression at public hospitals (Park et al., 2004). But, the other mechanism is uncertain now, and few studies have detailed the mediation effect of occupational stress between working hours and depression in China.

Former studies found the relationship between these variables separately, but few focused on the mediating effects of them. The study investigated the situation of long working hours for couriers in Zhejiang, China, and firstly investigated the effect of occupational stress and well-being on depression in couriers with long working hours. By constructing a SEM intermediary model, the mediating effects of occupational stress between working hours and depression were found, it provided important suggestions for the administrators to reduce the phenomenon of long working hours and take interventions targeting work-related stressors that will reduce the negative effects of depression among couriers. These results would seem to suggest that by reducing the incidence of occupational stress, depression that occurred by long working hours could effectively be reduced.

However, this study also has limitations. First, because this was a cross-sectional study, the cause-and-effect relationship between working hours, depression, occupational stress, and well-being could not be confirmed. Therefore, it is necessary for a longitudinal study to verify whether long working hours could have effects on depression, occupational stress, and well-being. Second, all the variables in the study were determined by scales, and laboratory indicators such as biomarkers were not used as the basis for diagnosis.

## Conclusion

It is common for couriers to work for a long time in Zhejiang, China. In addition, from the results of SEM, long working hours can lead to depression, occupational stress, and poor well-being. Working hours had a direct effect and indirect effect on depression *via* occupational stress, while occupational stress had a positive direct effect on depression. Therefore, decreasing working hours can effectively reduce the occurrence of depression and occupational stress. And it will be an effective way to reduce depression by decreasing working hours and preventing occupational stress.

## Data Availability Statement

The raw data supporting the conclusions of this article will be made available by the authors, without undue reservation.

## Ethics Statement

The studies involving human participants were reviewed and approved by National Institute of Occupational Health and Poison Control, Chinese Center for Disease Control and Prevention. The patients/participants provided their written informed consent to participate in this study.

## Author Contributions

HY: investigation and formal analysis. YZ: writing original draft and investigation. PX: review and editing and supervision. XF: investigation and data curation. LZ: methodology and investigation. FW: formal analysis. XL: conceptualization. HZ: funding acquisition. All authors contributed to the article and approved the submitted version.

## Funding

This research was funded by the Chinese Center for Disease Control and Prevention for the Special Investigation Project on Occupational Disease Hazards of key population (grant number 131031109000190002); the Zhejiang Provincial Program for Epidemiological Characteristics and Exposure risk of Musculoskeletal Injuries caused by adverse ergonomic factors in key industries (grant number 2019KY056); the Chinese Center for Disease Control and Prevention for the occupational Health Risk Assessment and International Occupational Health Standards Formulation (grant number 131031109000160004); and 2021 Zhejiang Province Center for Disease Control and Prevention for the Technology Talent Incubation Project.

## Conflict of Interest

The authors declare that the research was conducted in the absence of any commercial or financial relationships that could be construed as a potential conflict of interest.

## Publisher’s Note

All claims expressed in this article are solely those of the authors and do not necessarily represent those of their affiliated organizations, or those of the publisher, the editors and the reviewers. Any product that may be evaluated in this article, or claim that may be made by its manufacturer, is not guaranteed or endorsed by the publisher.
