# Supplementary material for: The Influence of Long Working Hours, Occupational Stress, and Well-Being on Depression Among Couriers in Zhejiang, China
Source: Front Psychol. 2022 Jun 23;13:928928. doi: 10.3389/fpsyg.2022.928928 (PMC9260285; doi:10.3389/fpsyg.2022.928928)
Supplement: Supplementary file 1 [file Data_Sheet_1.PDF]

Table1- Relationship between working hours and depression

Working hours	Model 1<br>Depression	Model 2<br>Depression	Model 3<br>Depression
≤48h	1	1	1
49-62h	1.13（0.78~1.64）	1.10（0.71~1.69）	1.12（0.73~1.72）
P	0.514	0.672	0.612
63-77h	1.76（1.25~2.50）	1.79（1.78~2.72）	1.86（1.22~2.83）
P	0.001	0.006	0.004
78-92h	1.75（1.18~2.6）	1.81（1.13~2.90）	1.94（1.21~3.12）
P	0.005	0.014	0.006
≥93	2.84（1.57~5.13）	4.29（2.12~8.67）	1.17（1.88~7.65）
P	0.001	<0.001	<0.001

Note: Model 1: Binary Logistic regression analysis;
Model 2: Multivariate Logistic regression analysis, adjusted for the different levels of occupational stress;
Model 3: Multivariate Logistic regression analysis, adjusted for whether had poor well-being.
